# RST-Net: A Semantic Segmentation Network for Remote Sensing Images Based on a Dual-Branch Encoder Structure

**DOI:** 10.3390/s25175531

**Published:** 2025-09-05

**Authors:** Na Yang, Chuanzhao Tian, Xingfa Gu, Yanting Zhang, Xuewen Li, Feng Zhang

**Affiliations:** 1 College of Remote Sensing and Information Engineering, North China Institute of Aerospace Engineering, Langfang 065000, China; 19935342291@163.com (N.Y.); zhangyanting0309@163.com (Y.Z.); 15192839463@163.com (X.L.); zhangfeng202303@163.com (F.Z.); 2 Collaborative Innovation Center of Aerospace Remote Sensing Information Processing and Application of Hebei Province, Langfang 065000, China; 3 Aerospace Information Research Institute, Chinese Academy of Sciences, Beijing 100094, China

**Keywords:** dual-branch encoder structure, remote sensing images, semantic segmentation, multi-scale feature fusion

## Abstract

High-resolution remote sensing images often suffer from inadequate fusion between global and local features, leading to the loss of long-range dependencies and blurred spatial details, while also exhibiting limited adaptability to multi-scale object segmentation. To overcome these limitations, this study proposes RST-Net, a semantic segmentation network featuring a dual-branch encoder structure. The encoder integrates a ResNeXt-50-based CNN branch for extracting local spatial features and a Shunted Transformer (ST) branch for capturing global contextual information. To further enhance multi-scale representation, the multi-scale feature enhancement module (MSFEM) is embedded in the CNN branch, leveraging atrous and depthwise separable convolutions to dynamically aggregate features. Additionally, the residual dynamic feature fusion (RDFF) module is incorporated into skip connections to improve interactions between encoder and decoder features. Experiments on the Vaihingen and Potsdam datasets show that RST-Net achieves promising performance, with MIoU scores of 77.04% and 79.56%, respectively, validating its effectiveness in semantic segmentation tasks.

## 1. Introduction

Semantic segmentation of remote sensing images (RSI), an interdisciplinary topic between computer vision and geographic information science, targets the pixel-wise classification of land cover to facilitate fine-scale interpretation and analysis. Enabled by advancements in high-resolution satellite imagery and multi-modal sensing technologies, RSI semantic segmentation has been widely applied in land resource monitoring [1,2], disaster response [3,4], and urban infrastructure planning [5,6]. Traditional methods like support vector machines (SVM) [7] and decision trees [8] depend heavily on hand-crafted spectral and texture features, limiting their effectiveness to low-resolution images. However, they struggle to capture the spectral–spatial correlations essential for accurate modeling in high-resolution images.

The rise of deep learning has established the convolutional neural network (CNN) as the predominant approach for semantic segmentation, facilitating automated hierarchical feature abstraction from low-level visual patterns to high-level semantic representations. Since the introduction of the fully convolutional networks (FCN) [9], a wide range of derivative structures have been developed to enhance segmentation performance. For instance, the SDFCNv1 framework [10] enhances feature fusion using dense skip connections, while SDFCNv2 [11] further extends the receptive field and reduces parameter overhead, thus improving segmentation efficiency. Predominantly adopting an encoder–decoder structure, current methods utilize the encoder for deep semantic feature extraction, with the decoder restoring spatial details progressively via skip connections and upsampling operations. Owing to its symmetric encoder–decoder structure and effective cross-layer feature propagation, U-Net [12] has become a prevalent choice for RSI segmentation. SegNet [13] enhances feature reconstruction by retaining max-pooling indices from the encoder to guide the decoder’s upsampling process. PSPNet [14] incorporates a spatial pyramid pooling (SPP) module to improve the fusion of multi-scale contextual features. The DeepLab family [15,16,17] utilizes atrous spatial pyramid pooling (ASPP), a mechanism designed to expand receptive fields while aggregating multi-scale contextual features. Despite their efficacy in natural image segmentation, these methods exhibit restricted applicability to RSI owing to the inability of fixed-size convolutional kernels to accommodate varying object scales. To address this, various multi-branch convolutional structures have been proposed to enhance multi-scale feature representation. For example, HRNet [18] maintains multi-resolution representations via parallel convolution streams, enabling the preservation of fine-grained spatial details and substantially improving the segmentation accuracy of linear features such as roads and rivers. RefineNet [19] refines feature map fusion via multi-path refinement modules, thereby improving the detection of small-scale targets and object boundaries. ResNeXt [20] extends ResNet [21] by introducing the concept of “cardinality”, which enhances feature diversity via parallel convolution branches and achieves improved scale adaptability without increasing computational cost.

In addition, attention mechanisms have been incorporated into CNN to improve feature discriminability and contextual representation. SE-Net [22] explicitly models inter-channel dependencies via squeeze-and-excitation modules, allowing adaptive recalibration of channel-wise responses. Attention U-Net [23] introduces a spatial attention gating mechanism to dynamically emphasize salient regions. CBAM [24] refines feature representation by jointly applying channel and spatial attention, while DANet [25] similarly employs dual attention to capture long-range dependencies. Based on the self-attention mechanism, the Transformer [26] significantly improves global context perception by modeling long-range pixel-wise dependencies. For instance, Lu et al. proposed a regularized Transformer [27] with adaptive token fusion for Alzheimer’s disease diagnosis in brain magnetic resonance imaging, dynamically selecting and fusing informative image tokens while regularizing feature diversity, and introduced a large adaptive filter and report-guided multi-level alignment network [28] in chest X-rays, leveraging textual reports for weakly supervised alignment. Moreover, CTBViT [29], a ViT variant with an efficient block design and randomized classifier for tuberculosis classification, has been proposed to enhance feature efficiency and generalization. Vision Transformer [30] pioneered the division of images into sequential patches, yet its global attention incurs quadratic complexity with image size, limiting its scalability for high-resolution RSI. Swin Transformer [31] mitigates computation via local window-based attention, but the fixed window size hampers the continuity of elongated structures such as roads and rivers. Shunted Transformer [32] introduces a dynamic receptive field mechanism, allowing each attention head to adaptively attend to spatial regions at multiple scales. While retaining linear computational complexity, it effectively enhances multi-scale feature representation, particularly under complex urban conditions involving vegetation and dense built-up areas. Transformer-based structures have demonstrated superior global consistency over CNN in tasks like farmland boundary delineation and urban structure extraction. However, pure Transformer models lack inherent local inductive bias, making them less sensitive to fine-grained textures and computationally inefficient for high-resolution images. Owing to the complementary capabilities of CNN in local feature extraction and Transformers in global contextual modeling, their integration has become a prevailing strategy for advancing segmentation performance. For instance, TransUNet [33] enhances global semantic consistency by embedding Transformer layers into the encoder; however, its single-path encoder structure is insufficient for preserving high-frequency spatial details. Swin-UNet [34] improves segmentation accuracy in remote sensing images using window-based self-attention, but its fixed window size limits the ability to dynamically model objects at varying scales. RSFormer [35] introduces a dual-branch spectral–spatial attention mechanism to optimize multi-modal feature fusion, yet its edge reconstruction performance on high-resolution images remains suboptimal. Although these hybrid CNN–Transformer structures have achieved promising results in remote sensing semantic segmentation, they still fall short in capturing the complex nonlinear interactions between local detail features and global semantic representations, thereby limiting their adaptability to objects of diverse scales.

To overcome these limitations, we present RST-Net, a novel semantic segmentation network with an encoder–decoder structure. The encoder adopts a dual-branch design that combines the strengths of CNN and Transformer to capture multi-scale features. A multi-scale feature enhancement module (MSFEM) is embedded in the CNN branch to enrich the feature representation. Moreover, a residual dynamic feature fusion (RDFF) module is introduced into skip connections to adaptively align and fuse multi-scale encoder features with high-level decoder semantics, thereby optimizing the feature transmission pathway and enhancing segmentation performance.

The core contributions of this study are as follows:

1. We propose a novel dual-branch encoder that integrates a ResNeXt-50-based CNN and a Transformer in parallel, effectively combining local detail extraction and global context modeling to generate rich multilevel feature representations.
2. We design the MSFEM that integrates various convolution operations, such as atrous and depthwise separable convolutions, to dynamically extract and aggregate multi-scale features, thereby addressing the limitations of inadequate multi-scale representation.
3. We introduce the RDFF module to alleviate semantic–detail conflicts in conventional skip connections by integrating residual connections with channel–spatial dual attention, enabling the adaptive fusion of deep semantic and shallow detail features.

## 2. Related Work

### 2.1. Remote Sensing Image Semantic Segmentation

Remote sensing image semantic segmentation is a pixel-wise classification task that leverages deep learning to enable accurate and automated identification of land cover types, thereby supporting a wide range of remote sensing applications. Over the past decade, a series of deep learning-based architectures have been developed to enhance segmentation accuracy and efficiency. FCN pioneered the application of end-to-end deep learning in semantic segmentation, serving as a foundational milestone. Since then, various architectures have been developed to improve segmentation performance across diverse remote sensing scenarios. Among them, U-Net effectively captures multi-scale contextual information via a symmetric encoder–decoder structure and skip connections, enhancing boundary localization accuracy. SegNet innovatively introduces pooling indices to optimize upsampling and reduce computational burden. Despite these advancements, the limited receptive field of conventional CNN impedes the modeling of long-range dependencies. This limitation reduces segmentation accuracy in complex scenes with multi-scale objects. To address this, numerous multi-scale feature fusion strategies have been explored. For example, DeepLabv3+ employs ASPP to extract multi-scale context. Similarly, PSPNet aggregates global features using pyramid pooling. Nevertheless, these methods still rely on the localized perception of convolutional operations.

To overcome these localization constraints, attention mechanisms have been introduced to refine feature interactions via reweighting strategies. Wang et al. proposed ECANet [36], which integrates channel-wise attention with an efficient computation strategy to further improve model performance. Similarly, CCNet [37], developed by Huang et al., improves CNN’s ability to model spatial–channel relationships, facilitating better capture of contextual information across channels, scales, and orientations. Although attention mechanisms enable interaction between local and global features through reweighting, they remain limited by the fixed receptive fields of convolutional kernels. Therefore, explicit global dependency modeling is necessary. In this regard, Transformer models significantly improve segmentation consistency for large-scale features by dynamically constructing global context via inter-pixel relationships. Nonetheless, the quadratic computational complexity of self-attention with increasing image size restricts its use in real-time, high-resolution remote sensing applications.

Consequently, hybrid architectures synergizing CNN-driven local feature extraction and Transformer-based global context modeling have emerged. Representatively, TransUNet employs feature fusion to integrate both information types; however, deep Transformer stages may attenuate high-frequency spatial details. BANet [38] addresses this by introducing a bidirectional attention mechanism, leveraging convolutional branches to preserve fine-grained details while using Transformer branches to capture long-range dependencies. In contrast, UNetFormer [39] employs a CNN–Transformer hybrid architecture characterized by enhanced modularity, where CNN serves as the encoder and a Transformer functions as the decoder. CMTFNet [40] further enhances multi-scale global representation by incorporating multi-scale self-attention in an encoder–decoder architecture. These trends motivate our design of a parallel dual-branch encoder, which integrates CNN and Transformer branches to independently extract and subsequently fuse multilevel features, thereby synergizing localized detail representation and global contextual modeling capabilities.

### 2.2. Multi-Scale Feature Extraction

The core challenge in semantic segmentation of RSI lies in the considerable variation of object scales across spatial dimensions, necessitating the development of feature extraction networks capable of robust multi-scale representation. Early studies primarily concentrated on improving the structural capabilities of convolutional operations for multi-scale feature learning. For instance, the ASPP module in DeepLabv3+ constructs a cross-scale context aggregation framework via multi-rate atrous convolutions. Building upon this, Shang et al. [41] proposed the multi-scale context extraction module (MSCEM), which innovatively integrates atrous convolutions with varying dilation rates and global average pooling to achieve parallel multi-scale context feature extraction. With the advancement of feature fusion theory, the feature pyramid network (FPN) [42] was introduced to tackle the multi-scale feature representation problem by adopting a top-down pathway with lateral connections, enabling the construction of semantically rich multilevel feature maps. Subsequent improvements, such as Recursive-FPN [43] and BiFPN [44], have further enhanced the flow and depth of multi-scale information by leveraging recursive stacking and efficient bidirectional cross-scale pathways, thus improving scale-aware feature characterization. Despite their effectiveness, these approaches rely heavily on fixed-size convolutional kernels or static attention windows, which limit their adaptability to dynamic and heterogeneous object scales in complex scenes.

To overcome this limitation, attention has shifted toward dynamic and adaptive mechanisms. Wang et al. [45] introduced a multi-scale attention pyramid framework to adaptively enhance semantic regions using attention-driven feature selection. Similarly, Xiao et al. [46] achieved multilevel feature extraction via dynamically adjustable receptive fields using a window-based attention mechanism. Fan et al. [47] proposed the Multi-scale Vision Transformer (MViT), which hierarchically increases the channel capacity while progressively reducing the spatial resolution across stages. This design effectively integrates Transformer structures with multi-scale feature hierarchies. A seminal advancement in this domain was achieved by Ren et al. through their Shunted Transformer architecture, which incorporates a novel mixed-scale attention mechanism that enables concurrent processing of features at varying spatial resolutions. This innovative design substantially enhances the model’s capacity for capturing cross-scale contextual relationships. Building upon this foundation, Yu et al. [48] developed MS-TCNet, integrating a shunted transformer encoder with a pyramid-structured decoder to hierarchically extract and refine multi-scale features. Zhou et al. [49] subsequently advanced this paradigm by architecting a hybrid framework that synergistically combines the Shunted Transformer with a multi-scale convolutional attention network (MSCAN), specifically optimized for remote sensing image segmentation tasks. This design not only ensures segmentation accuracy but also reduces computational overhead. Building upon these advances, this study employs the Shunted Transformer as one of the encoder branches to leverage its unique mixed-scale self-attention (SSA) mechanism for efficient multi-scale feature extraction. In parallel, the MSFEM is embedded within the CNN branch. This module utilizes a content-adaptive dynamic aggregation strategy, enabling the network to flexibly recalibrate multi-scale feature representations based on input characteristics, ultimately enhancing segmentation performance in complex remote sensing scenarios.

### 2.3. Skip Connections

Skip connections were originally designed to address the challenge of vanishing gradients. For instance, ResNet introduced skip connections via cross-layer identity mapping but lacks sufficient flexibility for adaptive multi-scale feature enhancement. In contrast, DenseNet [50] extended this concept by densely connecting each layer to all preceding ones, thereby promoting feature reuse and gradient flow. However, its fixed concatenation pattern lacks robustness against background clutter in remote sensing images. UNet employs symmetric skip connections that help retain spatial detail. However, its fixed weighting strategy limits adaptability to ambiguous boundaries and hinders effective interaction between semantic-level features.

To accommodate the multi-scale characteristics of RSI, skip connections have progressively evolved into a core mechanism for feature fusion. FPN introduced a top-down pyramid structure that effectively addresses the challenge of fusing shallow spatial details with deep semantic features. HRNet employs a parallel multi-resolution stream structure with densely connected skip links to maintain high-resolution representations, significantly improving the segmentation accuracy of small objects. However, its complex multi-branch design increases computational overhead. MCAT-UNet [51] integrates cross-shaped window attention (CSWT) into skip connections, enabling self-attention computation within local windows to retain the long-range dependency modeling capability of Transformers while reducing computational complexity. UCTransNet [52] redefines skip connections in U-Net by introducing a channel Transformer (CTrans) module, which adaptively bridges semantic gaps between encoder and decoder features, achieving precise image segmentation through enhanced global context modeling. However, its high computational cost hinders seamless integration into other network structures. The dynamic feature fusion (DFF) [53] module adaptively weights and fuses local feature maps based on global information, demonstrating advantages in feature selection. However, it suffers from two limitations: excessive reliance on global statistics may result in the loss of local contextual information, and the lack of residual learning mechanisms constrains deep feature representation. To address these issues, we propose the RDFF module, which integrates residual connections and attention mechanisms. RDFF preserves the global modeling capability of DFF while enhancing spatial detail retention, thereby achieving effective fusion of deep semantic features from the encoder and shallow spatial features from the decoder.

## 3. Proposed Method

**Figure 1:**
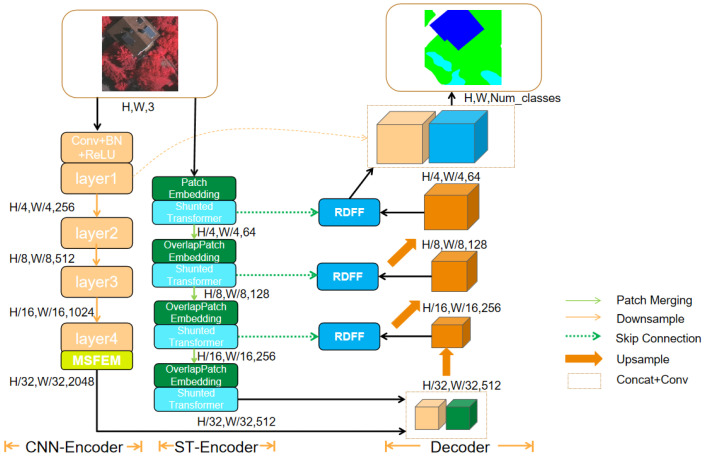
Overall structure of RST-Net, which consists of a dual-branch encoder structure composed of a CNN branch and an ST branch, a decoder, as well as the MSFEM and the RDFF module.

This study proposes a novel model, RST-Net, based on an encoder–decoder structure. The overall framework is illustrated in Figure 1. The encoder adopts a parallel dual-branch design, constructed based on ResNeXt-50 convolutional neural networks and the Shunted Transformer (ST), aiming to jointly extract local spatial details and global semantic information. Both branches adopt a four-stage progressive downsampling structure. In the final stage, the CNN branch incorporates the MSFEM, which enhances multi-scale representation by parallel processing with multi-scale convolutional kernels and feature fusion. The ST branch performs downsampling through linear embedding layers and captures multi-scale global context using the Shunted Self-Attention (SSA) mechanism. At the end of the encoder, features from both branches undergo deep interaction and fusion through deformable convolutions, producing a hierarchical representation that integrates local details with global semantics. The decoder takes the fused features as input and progressively restores spatial resolution via cascaded transposed convolutions. After each upsampling stage, an RDFF module adaptively integrates the corresponding features from the ST branch encoder with the current decoder features, effectively compensating for information loss and enhancing boundary accuracy. Finally, the refined decoder features, obtained through multi-scale feature fusion, are processed by a classifier to generate the final pixel-wise semantic segmentation map.

### 3.1. Dual-Branch Encoder Structure

To concurrently capture localized spatial details and global semantic contexts in RSI, this paper introduces a dual-branch encoder structure featuring parallel CNN and Transformer pathways. These complementary branches specialize in extracting fine-grained textural features and holistic contextual representations, respectively. Each branch includes four progressive stages that generate multi-scale feature maps with resolutions of 1/4, 1/8, 1/16, and 1/32 of the input image.

The CNN branch employs ResNeXt-50 as the backbone due to its superior performance on ImageNet and its ability to increase feature diversity via enhanced cardinality. The input image is processed through four convolutional stages with downsampling to generate multi-scale feature maps $F_{CNN}^{l}$, where $l\in \left\{1,2,3,4\right\}$.

The Transformer branch employs a hierarchical Shunted Transformer structure with flexible receptive fields to enhance global context modeling. Each stage consists of a feature embedding layer and the SSA module. In Stage 1, the Patch Embedding block is employed to downsample the input image to 1/4 of its original resolution and expand channels. The remaining three stages adopt an OverlapPatch Embedding block based on convolution to maintain spatial continuity and progressively increase channel dimensions.

**Figure 2:**
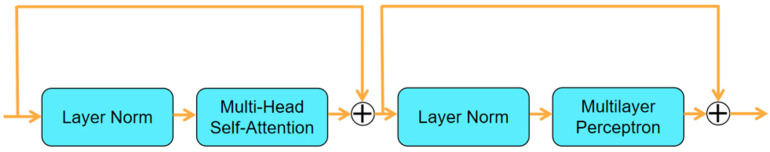
Structure of the Shunted Transformer Block, which is composed of MSA, MLP, and LN.

The SSA module is implemented via the Shunted Transformer Block, as shown in Figure 2, which integrates a Multi-scale Token Aggregation (MTA) mechanism to capture both local and global dependencies. The attention computation is as follows:

(1) $$Q_{i}=XW_{i}^{Q}$$

(2) $$K_{i},V_{i}=MTA\left(X,r_{i}\right)W_{i}^{K},MTA\left(X,r_{i}\right)W_{i}^{V}$$

(3) $$V_{i}=V_{i}+LE\left(V_{i}\right)$$

where $Q_{i}$, $K_{i}$, and $V_{i}$ represent the query, key, and value tensors of the ith head, $W_{i}^{Q}$, $W_{i}^{K}$, and $W_{i}^{V}$ are linear projection weights, $r_{i}$ is the downsampling ratio, and $LE\left(.\right)$ denotes the local enhancement module based on depthwise convolutions. Attention heads use varying downsampling rates $r_{i}$ to spatially compress K and V, followed by depthwise separable convolutions for local enhancement. The outputs from all heads are concatenated and passed through a residual feed-forward network to obtain the output feature maps $F_{ST}^{l}$, where $l\in \left\{1,2,3,4\right\}$.

At the cross-branch fusion stage, the output features $F_{CNN}^{4}$ and $F_{ST}^{4}$ from the fourth stage are concatenated along the channel dimension. To suppress redundancy, a 1×1 convolution compresses concatenated features from 1024 to 512 channels, while a 3×3 deformable convolution with adaptive spatial alignment dynamically corrects spatial misalignment between CNN and Transformer branches. Prioritizing high-response regions, this design synergistically enhances local textural details and global long-range dependencies to provide high-quality representations for the decoder.

### 3.2. Multi-Scale Feature Enhancement Module (MSFEM)

**Figure 3:**
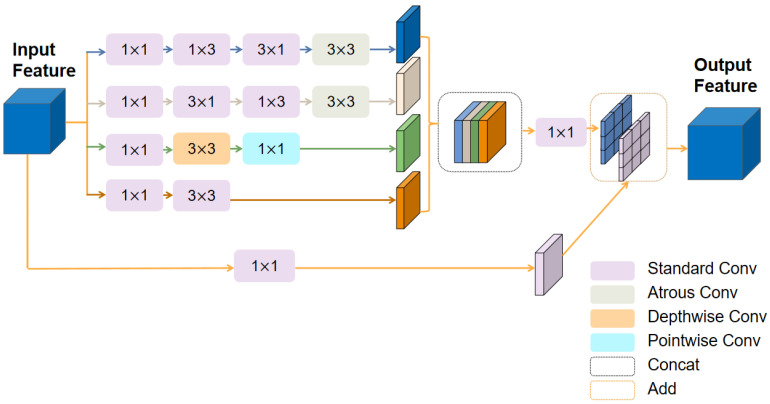
Structure of the MSFEM.

Traditional convolutional neural networks, constrained by their fixed receptive fields and static convolutional kernels, face challenges in effectively modeling multi-scale targets and complex spatial configurations. To address this issue, this study introduces the MSFEM. The module innovatively integrates heterogeneous convolutional architectures with learnable dynamic adjustment mechanisms, explicitly expanding the model’s receptive field scope while enabling adaptive feature re-weighting, fundamentally overcoming the static constraints of conventional convolutions. The structure of the MSFEM is illustrated in Figure 3.

Given an input feature map $X\in R^{H\times W\times 3}$, a four-branch parallel structure is employed for multi-scale feature extraction. The 1 × 1 convolution at the first layer of each branch is mainly used for channel compression and computational optimization. To capture local spatial details, a standard 3 × 3 convolution combined with a 1×1 convolution is utilized at branch 1 for feature extraction in the base space, thereby enhancing the characterization of local details:

(4) $$Q_{0}=ReLU\left({Conv}_{3\times 3}\left({Conv}_{1\times 1}\left(X\right)\right)\right)$$

where ${Conv}_{3\times 3}$ denotes 3×3 convolution and ${Conv}_{1\times 1}$ denotes 1×1 convolution. To capture large receptive field features and contextual information in both horizontal and vertical directions, complementary atrous convolution pyramids are constructed by cascading asymmetric convolutions and a 3×3 atrous convolution with a dilation rate of 5 in branches 2 and 3. This approach extracts directional features effectively:

(5) $$Q_{1}=ReLU\left({Conv}_{3\times 3}^{d=5}\left({Conv}_{3\times 1}\left({Conv}_{1\times 3}\left({Conv}_{1\times 1}\left(X\right)\right)\right)\right)\right)$$

(6) $$Q_{2}=ReLU\left({Conv}_{3\times 3}^{d=5}\left({Conv}_{1\times 3}\left({Conv}_{3\times 1}\left({Conv}_{1\times 1}\left(X\right)\right)\right)\right)\right)$$

where ${Conv}_{3\times 3}^{d=5}$ denotes a 3 × 3 atrous convolution with a dilation rate of 5. Branch 4 employs a depthwise separable convolution to enhance local feature sensitivity through channel-wise computations, allowing the network to dynamically focus on salient regions and perform spatially adaptive feature modulation:

(7) $$Q_{3}=ReLU\left(D{Conv}_{3\times 3}\left({Conv}_{1\times 1}\left(X\right)\right)\right)$$

where $D{Conv}_{3\times 3}$ denotes a 3 × 3 depthwise separable convolution. The outputs from the four branches, including micro-level detail $Q_{0}$, horizontal and vertical contextual features $Q_{1}$ and $Q_{2}$, as well as dynamic calibration features $Q_{3}$, are concatenated along the channel dimension to construct a rich multi-dimensional feature representation:

(8) $$U=Concat\left(Q_{0},Q_{1},Q_{2},Q_{3}\right)$$

where Concat denotes channel merging.

Finally, a 1×1 convolution is applied to facilitate cross-channel information interaction. Combined with a learnable scaling factor, the module performs adaptive weighted feature fusion:

(9) $$O={Conv}_{1\times 1}\left(U\times scale+{Conv}_{1\times 1}\left(X\right)\right)$$

where scale represents a learnable scaling factor that dynamically allocates the importance of each feature component. Here we set scale=0.1, which effectively balances the convergence speed and final accuracy required for the task.

### 3.3. Residual Dynamic Feature Fusion (RDFF)

Traditional skip connections often exhibit low feature fusion efficiency due to the inherent conflict between deep semantic features and shallow spatial details. The DFF module addresses this issue by adaptively learning feature importance and performing weighted fusion to improve segmentation accuracy. Inspired by this concept, we propose the RDFF module, which integrates encoder-derived semantic features with decoder-generated spatial details through a synergistic design of channel-spatial dual attention and residual learning.

**Figure 4:**
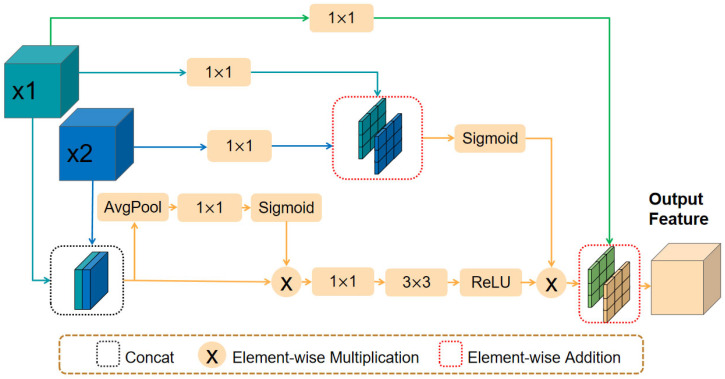
Structure of the RDFF module.

The RDFF module is embedded within the skip connections, where it integrates the upsampled shallow decoder features from the decoder with the deep encoder features from the corresponding stage of the ST branch. These features are fused via the RDFF module. The structure of the RDFF module is illustrated in Figure 4.

Given the shallow feature map $X_{1}\in R^{H\times W\times C}$ from the decoder and the deep feature map $X_{2}\in R^{H\times W\times C}$ from the encoder, both are first passed through separate 1×1 convolutions to align their channel dimensions. The transformed features are then concatenated along the channel dimension.

(10) $$U=Concat\left({Conv}_{1\times 1}\left(X_{1}\right),{Conv}_{1\times 1}\left(X_{2}\right)\right)$$

where Concat denotes the concatenation operation along the channel dimension, and ${Conv}_{1\times 1}$ denotes the 1×1 convolution operation. Subsequently, global average pooling is used to extract global information, and a 1×1 convolution is used to compute the channel attention weights of the combined feature map, which are then applied to the combined feature map.

(11) $$A_{c}=\sigma \left({Conv}_{1\times 1}\left(AvgPool\left(U\right)\right)\right)$$

(12) $$U^{\prime }=U⊗A_{c}$$

where σ denotes the Sigmoid activation function, AvgPool denotes adaptive average pooling, and ⊗ denotes element-wise multiplication. To enhance feature representation capabilities and capture broader contextual information, a 1×1 convolution is further applied to the weighted feature map for channel dimension reduction, followed by a 3×3 convolution layer for spatial feature extraction.

(13) $$V=ReLU\left({Conv}_{3\times 3}\left({Conv}_{1\times 1}\left(U^{\prime }\right)\right)\right)$$

where ${Conv}_{3\times 3}$ denotes the 3×3 convolution operation. To adaptively adjust the spatial weights of features from different input layers, a spatial attention mechanism is introduced. Spatial attention weights are computed via 1×1 convolutions. The two attention weights are then summed and normalized to obtain the spatial attention weight.

(14) $$A_{s}=\sigma \left({Conv}_{1\times 1}\left(X_{1}\right)+{Conv}_{1\times 1}\left(X_{2}\right)\right)$$

Finally, a residual connection is employed to combine the spatially attended features with the original shallow feature map, ensuring feature integrity.

(15) $$O=V⊗A_{s}+{Conv}_{1\times 1}\left(X_{1}\right)$$

## 4. Experiments

### 4.1. Datasets

To holistically evaluate model efficacy, we conduct experimental validation on the ISPRS-released Vaihingen and Potsdam benchmark datasets. These publicly available datasets encompass six semantic classes: impervious surfaces, buildings, low vegetation, trees, cars, and background.

The Vaihingen dataset encompasses urban regions of Vaihingen, Germany, comprising 33 orthorectified aerial images at 9 cm spatial resolution with heterogeneous spatial coverage. The images exhibit heterogeneous spatial coverage, averaging 2494 × 2064 pixels in size. Each image features near-infrared, red, and green spectral bands (IRRG), complemented by both digital surface models (DSMs) and normalized DSMs (nDSMs). This investigation designated 16 orthophotos (IDs 1–16) for training purposes, reserving the remaining 17 images (IDs 17–33) for testing procedures.

The Potsdam dataset spans the historic city center of Potsdam, Germany, and comprises 38 orthorectified images with a spatial resolution of 5 cm and consistent dimensions. All images have consistent dimensions of 6000 × 6000 pixels. The dataset provides images in both true-color (RGB) and IRRG formats, along with corresponding DSM and nDSM data. Compared to Vaihingen, the Potsdam dataset presents a denser urban fabric, characterized by large building complexes, narrow roadways, and intricate residential areas. In this study, 24 RGB-band images were selected for training, and the remaining 14 images (IDs: 2_13–7_13) were used for testing.

### 4.2. Experimental Settings

Experimental execution leveraged the PyTorch 1.13 framework with model construction and training conducted on an NVIDIA RTX A4000 GPU. The Ranger optimizer, featuring a 0.001 weight decay coefficient, was implemented to bolster convergence stability and counteract overfitting. Training spanned 100 epochs using a batch size of 16 alongside an initial 0.01 learning rate adaptively regulated through cosine annealing. Input images were standardized by random cropping into 256 × 256 pixel patches. Stochastic combinations of augmentation techniques containing random flipping, scaling, brightness and contrast adjustments enhanced model generalization.

To mitigate class imbalance in the Vaihingen and Potsdam datasets, the cross-entropy loss function is utilized, mathematically expressed as follows:

(16) $$L_{CE}=-\sum_{i=1}^{n}\log\left(\hat{y_{i}}\right)$$

where n denotes the number of classes, $y_{i}$ represents the true value, and $\hat{y_{i}}$ indicates the softmax probability of the i-th class.

### 4.3. Evaluation Metrics

To ensure a comprehensive and objective assessment, the present study employs Overall Accuracy (OA), Mean Intersection over Union (MIoU), and the F1 score as primary evaluation metrics for RSI semantic segmentation performance. These metrics evaluate model performance from multiple perspectives, encompassing overall classification accuracy, object boundary localization, and class-wise prediction balance. The mathematical formulations of these metrics are defined as follows:

(17) $$OA=\frac{\sum_{i=1}^{N}{TP}_{i}}{\sum_{i=1}^{N}\left({TP}_{i}+{FP}_{i}{+{TN}_{i}+FN}_{i}\right)}$$

(18) $$MIoU=\frac{1}{N}\sum_{i=1}^{N}\frac{{TP}_{i}}{{TP}_{i}+{FP}_{i}+{FN}_{i}}$$

(19) $${Precision}_{i}=\frac{{TP}_{i}}{{TP}_{i}+{FP}_{i}}$$

(20) $${Recall}_{i}=\frac{{TP}_{i}}{{TP}_{i}+{FN}_{i}}$$

(21) $${F1}_{i}=\frac{{2\times Precision}_{i}{\times Recall}_{i}}{{Precision}_{i}{+Recall}_{i}}$$

where N denotes the total number of target categories, and ${TP}_{i}$, ${FP}_{i}$, ${TN}_{i}$, and ${FN}_{i}$ correspond to the number of true positive, false positive, true negative and false negative samples for class i, respectively. ${F1}_{i}$ denotes the F1 score for class i, and ${Precision}_{i}$ and ${Recall}_{i}$ are the precision and recall values for class i, respectively.

### 4.4. Ablation Study

To comprehensively evaluate the contributions of the dual-branch encoder structure, MSFEM, and RDFF to the model’s performance, systematic ablation experiments were conducted on the Vaihingen dataset. Specifically, six different model configurations were constructed as detailed in Table 1. All experiments were performed under consistent hyperparameter and training settings, with analyses focusing on the following three aspects:

#### 4.4.1. Effectiveness of the Dual-Branch Encoder Structure

**Figure 5:**
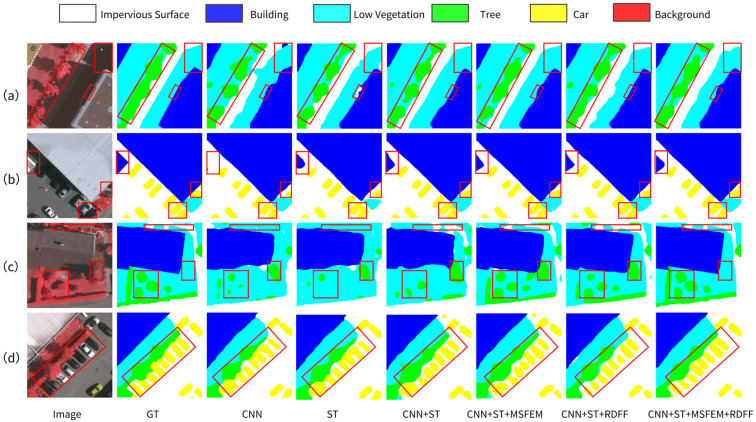
Visual results of ablation experiments on the Vaihingen dataset across four representative scenes. (**a**) Building and impervious surface integration. (**b**) Precise building boundary delineation with small car identification. (**c**) Hierarchical vegetation structure differentiation. (**d**) High-density car spatial partitioning. **CNN**: single-branch ResNeXt-50 model; **ST**: single-branch Shunted Transformer branch; **CNN+ST**: dual-branch model; **CNN+ST+MSFEM**: dual-branch with MSFEM; **CNN+ST+RDFF**: dual-branch with RDFF; **CNN+ST+MSFEM+RDFF**: full RST-Net with both modules. Red boxes highlight critical variation areas.

To validate the effectiveness of the dual-branch encoder structure, it was compared against single-branch CNN and single-branch ST models. As shown in the first three groups of Table 1, the dual-branch CNN+ST model significantly outperforms the single-branch counterparts, achieving OA, m-F1, and MIoU scores of 88.23%, 86.09%, and 76.06%, respectively. This corresponds to a 4.53% OA improvement over the single-branch CNN model, and MIoU and m-F1 gains of 2.66% and 1.73% over the single-branch ST model. As illustrated in Figure 5a, the CNN model exhibits severe fragmentation in elongated vegetation regions, primarily due to the loss of spatial resolution in deep layers. Conversely, the single-branch ST model captures global contextual dependencies but lacks sensitivity to fine-grained textures, leading to segmentation omissions around building boundaries. By integrating the local detail awareness of CNN with the global semantic modeling strength of Transformers, the proposed dual-branch structure achieves complementarity: it substantially mitigates vegetation fragmentation while more accurately delineating building boundaries. These results demonstrate that the dual-branch model effectively leverages the strengths of both CNN and Transformer, learning more discriminative feature representations that enhance semantic consistency and boundary delineation.

#### 4.4.2. Effectiveness of the MSFEM

To further assess the MSFEM, it was integrated into the dual-branch CNN+ST model alongside the RDFF module. Comparing the results between the third and fourth groups in Table 1, incorporating MSFEM improved the model’s MIoU to 76.40%, an increase of 0.34% over the dual-branch baseline. Notably, segmentation accuracy for low vegetation and trees increased by 1.30% and 2.76%, reaching 72.15% and 71.17%, respectively, indicating the module’s effectiveness in enhancing multi-scale morphological representation. Figure 5b shows that without MSFEM, the model struggles to distinguish tree from low vegetation in dense vegetation scenes due to fixed-scale convolution kernels, leading to blurred and missed segmentation. MSFEM’s multi-scale parallel convolutions capture hierarchical details from large-scale buildings to small-scale trees, significantly improving segmentation precision for heterogeneous land covers and enabling clear differentiation between small trees and low vegetation.

#### 4.4.3. Effectiveness of the RDFF Module

To verify the role of the RDFF module, it was also integrated into the dual-branch CNN+ST model. As shown in the third and fifth groups of Table 1, the addition of RDFF increased MIoU from 76.06% to 76.62%, and car IoU from 66.65% to 67.13%. Visualization in Figure 5d indicates severe adhesion issues in densely packed car areas when RDFF is absent. Incorporating RDFF effectively enhances local boundary features such as car contours through cross-level residual connections and channel-spatial dual attention mechanisms, significantly suppressing object adhesion. Figure 5c illustrates how RDFF leverages global average pooling to capture semantic consistency, combined with attention mechanisms to optimize local details, thereby alleviating boundary blurring in deep networks and reducing false positives in low vegetation areas.

In summary, the dual-branch encoder structure establishes the feature extraction foundation, the MSFEM module enhances multi-scale perception robustness, and the RDFF module resolves feature fusion conflicts through dynamic weighting. Their synergy enables the complete RST-Net model to achieve an MIoU of 77.04%, with Impervious Surface IoU exceeding 88.44%, and car IoU improving to 69.24%. Visualized segmentation results in Figure 5 clearly demonstrate that the full RST-Net yields superior edge clarity and category discrimination compared to partial-module configurations. The complete RST-Net model outperforms those containing only some modules by producing clearer edge details, effectively recognizing multi-scale land covers, and substantially reducing misclassification of individual objects.

### 4.5. Comparative Experiments

To systematically evaluate the performance of RST-Net, we compare it with several representative semantic segmentation approaches on the Vaihingen and Potsdam datasets, including classical CNN-based models (UNet [12], SegNet [13], DeepLabV3+ [16], PSPNet [14]) and recent Transformer-based structures (HST-Net [49], UNetFormer [39], and CMTFNet [40]). For fair comparison, all approaches are trained using the same experimental settings, data preprocessing pipeline, and augmentation strategies as RST-Net, and evaluated on identical training and test splits.

#### 4.5.1. Results on the Vaihingen Dataset

The quantitative results on the Vaihingen dataset are summarized in Table 2. As shown in Table 2, RST-Net consistently outperforms all baseline methods in overall performance. Specifically, RST-Net achieves an OA of 88.48%, an m-F1 of 86.77%, and a MIoU of 77.04%. Further analysis reveals that DeepLabV3+ underperforms in segmenting trees and low vegetation due to limited multi-scale representation. PSPNet, despite incorporating pyramid pooling for global context, still struggles with identifying small objects like cars. Although recent transformer-based models such as HST-Net, UNetFormer, and CMTFNet improve long-range dependency modeling and show gains across metrics, their overall performance remains inferior to RST-Net.

**Figure 6:**
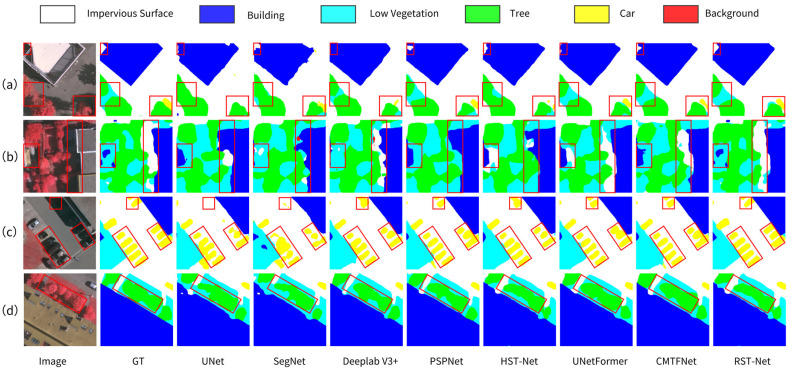
Visual results of comparative experiments on the Vaihingen dataset across four representative scenes. (**a**) Vegetation-tree-impervious surface integration. (**b**) Fine vegetation differentiation under shadow interference. (**c**) Spatial distribution analysis of high-density cars. (**d**) Structural separation of trees and low vegetation. Red boxes highlight critical variation areas.

Figure 6 presents visual segmentation results for four representative scenes, further demonstrating the advantages of RST-Net. Experimental results indicate that the UNet model suffers from significant blurring at the boundaries of various land-cover classes and especially underperforms in segmenting small objects. For example, missed car detections are observed in the lower-right corner of Figure 6a and the upper region of Figure 6c. The SegNet model shows confusion in distinguishing between impervious surfaces and vegetation areas, which is particularly evident in Figure 6b. The DeeplabV3+ model, by introducing dilated convolutions to enlarge the receptive field, achieves significantly improved classification accuracy compared to UNet and SegNet. However, it still suffers from under-segmentation and over-segmentation issues, failing to effectively address the adhesion between adjacent car targets. The PSPNet model alleviates the adhesion problem through a multi-scale feature fusion strategy. Compared to classical segmentation approaches, recent semantic segmentation models demonstrate substantially improved capability in distinguishing between different land-cover categories and significantly reduce misclassification rates. Nevertheless, segmentation deviations persist in complex scenes, making it difficult to accurately reconstruct the true spatial distribution. The proposed RST-Net model exhibits strong performance across various land-cover segmentation tasks, with notable advantages in both detail preservation and overall segmentation accuracy, thereby validating its effectiveness in semantic understanding under complex remote sensing scenarios.

#### 4.5.2. Results on the Potsdam Dataset

The quantitative comparison results on the ISPRS Potsdam dataset are presented in Table 3. The experimental results indicate that the proposed RST-Net achieves superior overall performance. Although the OA of RST-Net is slightly lower than that of CMTFNet by 0.51%, reaching 87.24%, it achieves the highest scores in both m-F1 and MIoU. Specifically, RST-Net achieves a MIoU of 79.56%, surpassing the second-best method, CMTFNet, by 1.53 percentage points. Its m-F1 reaches 88.51%, exceeding UNetFormer by 1.20 percentage points. A detailed category-wise analysis shows that RST-Net attains an IoU of 89.73% for buildings, outperforming UNet by 0.74 percentage points. For the car class, it achieves an IoU of 79.53%, surpassing SegNet by 3.19 points. Notably, for the tree class, RST-Net reaches 76.23% IoU, which is 8.31 points higher than DeeplabV3+.

**Figure 7:**
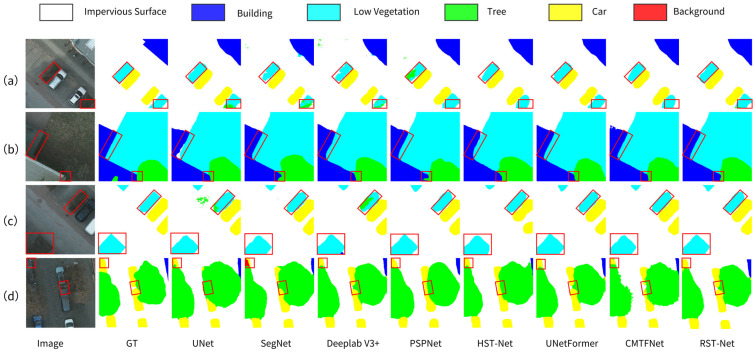
Visual results of comparative experiments on the Potsdam dataset across four representative scenes. (**a**) Inter-class differentiation of cars and low vegetation. (**b**) Precise boundary delineation between buildings and low vegetation. (**c**) Fine-grained edge refinement within low vegetation. (**d**) Car recognition under tree occlusion. Red boxes highlight critical variation areas.

The visual results on the Potsdam dataset, shown in Figure 7, further confirm RST-Net’s enhanced segmentation performance across various complex urban scenes. Compared to other models, RST-Net yields more precise segmentation results. Specifically, for distinguishing low vegetation from trees in Figure 7a,c, classical models like UNet exhibit notable misclassification due to spectral similarity, whereas recent methods such as HST-Net and UNetFormer improve classification accuracy but still face challenges in accurately delineating boundaries. By leveraging multi-scale feature fusion, RST-Net effectively distinguishes between low vegetation and trees, achieving higher segmentation accuracy than Deeplab V3+ and PSPNet. For the car segmentation task shown in Figure 7d, several comparative models suffer from misclassification caused by spectral mixing under tree canopies. RST-Net effectively addresses this issue through refined boundary modeling, reducing object adhesion and enhancing contour completeness. For building segmentation, RST-Net significantly preserves the geometric integrity of building boundaries, accurately reconstructing the spatial morphology and extent of real-world buildings. Overall, these results validate the effectiveness and superiority of RST-Net in multi-category semantic segmentation tasks for high-resolution RSI.

#### 4.5.3. Efficiency Analysis

Practical remote sensing deployments critically depend on model efficiency. Table 4 summarizes the number of parameters, computational complexity (FLOPs), speed (FPS), and segmentation performance (MIoU) of each model on the Potsdam dataset. Compared with the conventional model DeepLabV3+, which contains 122.01 MB of parameters, requires 52.21 GFLOPs of computation, and runs at 41.87 FPS, RST-Net achieves superior resource utilization and speed. Specifically, RST-Net reduces the parameter count by 53.2% to 57.17 MB and decreases computational complexity by 34.7% to 34.11 GFLOPs, while simultaneously achieving a significantly higher frame rate of 75.20 FPS, which is 79.6% faster than DeepLabV3+. Despite this reduction, it improves the MIoU by 4.83 percentage points, reaching 79.56%. Although the UNetFormer model presents a lightweight design with 11.7 MB of parameters and 11.14 GFLOPs of computation, achieving the highest FPS of 115.42, its MIoU score reaches only 77.69 percent, which is still 1.87 percentage points lower than that of RST-Net. The results indicate that RST-Net achieves a well-balanced optimization between segmentation accuracy and computational efficiency within a manageable range of parameters and operations, providing a practical solution for real-time analysis of high-resolution RSI.

## 5. Discussion

The proposed RST-Net demonstrates significant performance advantages in RSI, attributable to three core technical innovations. The dual-branch architecture leverages complementary CNN and Transformer designs to preserve local details while capturing global semantics. Concurrently, the MSFEM addresses the spectral variations within the same category challenge in RSI through parallel atrous convolutions and depthwise separable convolutions, enabling adaptive recognition of targets across scales. Furthermore, the RDFF mechanism optimizes hierarchical feature alignment via residual connections and substantially mitigates ground object adhesion through spatial attention-based weight redistribution.

However, this study exhibits limitations: the dataset exclusively covers German urban areas without validation in extreme environments. Additionally, the dual-branch encoder and attention mechanisms incur higher computational costs than lightweight CNN models, potentially compromising real-time performance on edge devices or during large-scale satellite image processing. Future work will integrate multispectral and SAR data to enhance classification robustness while developing dynamic network pruning strategies to accelerate inference without sacrificing accuracy.

## 6. Conclusions

This paper proposes a novel semantic segmentation structure, RST-Net, to overcome the limitations of global-local feature fusion and limited adaptability to multi-scale object segmentation in RSI. The network adopts a dual-branch encoder structure, combining a ResNeXt-50-based CNN branch and a Shunted Transformer branch to effectively extract local details and capture global context. To enhance multi-scale representation, the proposed MSFEM dynamically aggregates features at different scales, significantly improving the model’s responsiveness to spatial variability. Furthermore, the RDFF module facilitates seamless fusion between semantic and detail features across network stages through a coordinated attention mechanism.

Extensive experiments conducted on the ISPRS Vaihingen and Potsdam datasets validate the effectiveness of RST-Net, which achieves MIoU scores of 77.04% and 79.56%, respectively. Compared to DeepLabV3+, a representative encoder–decoder model, RST-Net achieves reductions of 53.2% in model size and 34.7% in computational complexity, while maintaining competitive segmentation accuracy.

Subsequent research efforts will aim to incorporate lightweight techniques, including but not limited to model pruning and knowledge distillation. This integration seeks to achieve further model compression and enhanced computational efficiency, thereby addressing the computational constraints inherent in real-time processing of high-resolution RSI.

## Figures and Tables

**Table 1:** Ablation study results on the Vaihingen dataset.

Method	IoU (%)					Evaluation Index		
	Impervious Surface	Building	Low Vegetation	Tree	Car	OA (%)	m-F1 (%)	MioU (%)
CNN	82.20	84.48	70.31	69.24	58.77	83.70	84.05	73.00
ST	81.83	84.22	70.60	70.27	60.08	86.39	84.36	73.40
CNN+ST	84.55	89.85	70.85	68.41	66.65	88.23	86.09	76.06
CNN+ST+MSFEM	84.19	89.98	72.15	71.17	64.51	88.47	86.31	76.40
CNN+ST+RDFF	84.37	89.85	71.65	70.13	67.13	88.40	86.49	76.62
CNN+ST+MSFEM+RDFF	88.44	90.20	71.61	69.69	69.24	88.48	86.77	77.04

**Table 2:** Comparison of semantic segmentation results on the Vaihingen dataset.

Method	IoU (%)					Evaluation Index		
	Impervious Surface	Building	Low Vegetation	Tree	Car	OA (%)	m-F1 (%)	MioU (%)
UNet [12]	82.20	84.48	70.31	69.24	58.77	83.70	84.05	73.00
SegNet [13]	81.83	87.33	69.20	67.35	59.21	86.80	83.98	72.98
DeeplabV3+ [16]	81.61	87.48	68.54	67.81	58.35	86.65	83.81	72.76
PSPNet [14]	82.32	86.88	69.65	69.39	61.34	87.08	84.68	73.92
HST-Net [49]	82.33	87.94	70.01	68.26	62.38	87.27	84.84	74.18
UNetFormer [39]	83.04	88.24	69.98	69.11	64.59	84.53	85.41	74.99
CMTFNet [40]	84.17	89.80	70.50	68.83	62.34	88.04	85.41	75.13
RST-Net (Ours)	88.44	90.20	71.61	69.69	69.24	88.48	86.77	77.04

**Table 3:** Comparison of semantic segmentation results on the Potsdam dataset.

Method	IoU (%)					Evaluation Index		
	Impervious Surface	Building	Low Vegetation	Tree	Car	OA (%)	m-F1 (%)	MioU (%)
UNet [12]	76.22	88.99	70.11	71.93	68.50	85.21	85.62	75.15
SegNet [13]	75.90	85.89	69.45	71.37	76.34	84.68	86.11	75.79
DeeplabV3+ [16]	75.38	86.59	68.96	67.92	74.79	84.05	85.38	74.73
PSPNet [14]	76.41	86.46	70.37	71.30	74.83	84.79	86.16	75.86
HST-Net [49]	76.84	87.28	70.99	71.50	76.61	85.28	86.66	76.64
UNetFormer [39]	77.94	88.65	71.09	72.28	78.00	85.89	87.31	77.69
CMTFNet [40]	77.05	87.54	72.18	74.79	78.57	87.75	87.56	78.03
RST-Net (Ours)	78.60	89.73	73.69	76.23	79.53	87.24	88.51	79.56

**Table 4:** Comparison of Params, FLOPs, FPS, and MIoU on the Potsdam dataset.

Method	Parameters (MB)	FLOPs (G)	MioU (%)	FPS
UNet [12]	23.89	18.85	75.15	87.35
SegNet [13]	80.63	28.08	75.79	60.12
DeeplabV3+ [16]	**122.01**	52.21	74.73	41.87
PSPNet [14]	92.45	44.54	75.86	46.05
HST-Net [49]	28.03	22.83	76.64	90.28
UNetFormer [39]	11.7	**11.14**	77.69	115.42
CMTFNet [40]	28.67	17.14	78.03	100.15
RST-Net (Ours)	57.17	34.11	**79.56**	**75.20**

## Data Availability

The original contributions presented in this study are included in the article. The source code and trained models for RST-Net will be made publicly available upon acceptance at https://github.com/white-yang1/RST-Net.git (accessed on 2 September 2025).
